# Frontal Mucopyocoele Presenting With Recurrent Periorbital Cellulitis: A Delayed Diagnosis

**DOI:** 10.7759/cureus.32252

**Published:** 2022-12-06

**Authors:** Javier Ash, Noor Omar, Victoria Perkins, Martyn Barnes

**Affiliations:** 1 Otolaryngology, Southend University Hospital, Southend-on-Sea, GBR; 2 Medicine, Anglia Ruskin University Medical School, Chelmsford, GBR

**Keywords:** mucopyocoele, orbital proptosis, orbital cellulitis, frontal mucocele, acute sinusitis

## Abstract

Periorbital cellulitis is an uncommon presentation to primary care and the emergency department. With multiple aetiologies, it is crucial that an appropriate history and examination are applied to identify the primary cause and initiate therapy in a timely manner.

We present a 30-year-old male who presented with recurrent periorbital cellulitis treated repeatedly with antibiotics without consideration of the origin of the infection. Subsequent investigations discovered a widely dehiscent mucopyocoele of the frontal sinus that had been unrecognised and untreated. Once identified, the patient underwent functional endoscopic sinus surgery to clear the mucopyocoele and improve the drainage of the frontal sinus. Symptoms have not recurred since the surgical intervention.

A poor understanding of the aetiologies of periorbital cellulitis and the related anatomy likely played a role in his delayed definitive management. Clinicians should be aware that in a patient presenting with periorbital swelling and erythema, consideration should be given to the possibility of underlying sinonasal pathology.

## Introduction

Periorbital cellulitis (also called pre-septal cellulitis) is an acute infection of the periorbital soft tissues, largely the eyelids, skin and soft tissues anterior to the orbital septum [1]. Patients present with unilateral eyelid swelling and oedema [2]. The infection can start from a primary skin, conjunctival or sinus origin, and may in turn progress beyond the orbital septum posteriorly as orbital cellulitis.

The paranasal sinuses surround the orbit and the knowledge of the anatomy of the area are important. In those infections arising from the sinuses, the ethmoid sinuses are the most common origin, with the infection spreading through the thin bone of the lamina papyracea to enter either anteriorly as pre-septal (periorbital) cellulitis or posteriorly as the more dangerous post-septal orbital cellulitis [3]. Frontal or maxillary sinusitis can also lead to similar presentations [4].

Chronic inflammatory disease of the paranasal sinuses can lead to nasal polyp and mucocoele formation. Mucocoeles are accumulations of mucoid secretions within a sinus due to the obstruction of the sinus ostium in the presence of ongoing mucous production [5,6]. The obstruction may result from inflammatory mucosal thickening, anatomical abnormalities and other causes of failure in the mucociliary escalator [5].

Around 60%-90% of all sinonasal mucocoeles are in the frontal sinus [7], with the involvement of the sphenoid, ethmoid and maxillary sinuses being rare [8]. Mucocoeles can behave like space-occupying lesions with bone erosion and displacement of surrounding structures [5].

Without adequate treatment, periorbital cellulitis of any origin can progress to true orbital cellulitis or the formation of abscesses, either periorbital, subperiosteal or orbital, and can (in extreme cases) lead to cavernous sinus thrombosis or intracranial extension of infection [1,9]. It is therefore important to ensure that a thorough history and examination of the patient are performed to elucidate the likely cause, as well as any evident complications. In recurrent cases (even with apparently uncomplicated periorbital cellulitis), computed tomography (CT) imaging is advisable.

The extent of an orbital infection, as well as any associated complications, can be described using Chandler’s classification [4]. Stage I (pre-septal) is cellulitis with inflammation and oedema anterior to the orbital septum. Stage II (post-septal) extends beyond the septum. Stage III is a (subperiosteal) collection beneath the periorbita (the periosteum within the bony margins of the orbit), usually stripping this from the lamina papyracea following the spread from the adjacent ethmoid sinuses. Stage IV is an orbital collection (inside the periorbita, within the post-septal orbital tissues, largely the fat). Stage V is a cavernous sinus thrombosis after the extension of infection via the superior ophthalmic veins.

It should be noted that although the classification is very useful in describing cases, the ‘stage’ terminology is misleading; there is by no means a stepwise continuity of severity, and the ‘stages’ might be better understood as ‘types’ of orbital cellulitis presentations, based on the location of the cellulitis, and associated complications. For example, a cavernous sinus thrombosis ‘stage V’ often has no preceding ‘stage’ III or IV period.

Both periorbital and orbital cellulitis can pose additional difficulties for clinicians due to its multi-speciality aetiology. Commonly, it is managed by ophthalmology, otolaryngology, emergency medicine, oral and maxillofacial surgery or paediatrics. Otolaryngologists can help determine the aetiology and guide the differential management of the condition, especially if a sinogenic cause is suspected [1].

Terminology is an additional barrier; for the current paper and in general, we feel that ‘periorbital cellulitis’ and ‘orbital cellulitis’ should be preferred to the more traditional (pre-septal and post-septal orbital cellulitis) terminology.

In this case report, we present a patient with a history of recurrent periorbital cellulitis, eventually found to be secondary to a mucopyocoele (infected mucocoele) arising from the frontal sinus, which failed to fully resolve with medical management until the sinus pathology had been addressed.

## Case presentation

A 30-year-old male attended the emergency department with a two-month history of intermittent swelling and erythema affecting the right eye. The patient was systemically well with no history of trauma and no relevant past medical history or allergies. He had been previously assessed and diagnosed with suspected periorbital cellulitis and managed with appropriate oral and topical antibiotics; however, this had only temporarily resolved his symptoms on each occasion.

Prior to the onset of swelling, he reported a severe headache with sharp throbbing pain localised over the right eye. Over the subsequent 24 hours, his right periorbital region and both (upper and lower) eyelids became swollen and erythematous with chemosis and associated watery discharge. Eye opening was restricted, but there was no ophthalmoplegia. Pupillary reflexes, visual acuity and colour vision were normal. There was no relative afferent pupillary defect (RAPD). The patient was reviewed by ophthalmology who diagnosed periorbital cellulitis and commenced further oral antibiotics.

**Figure 1:**
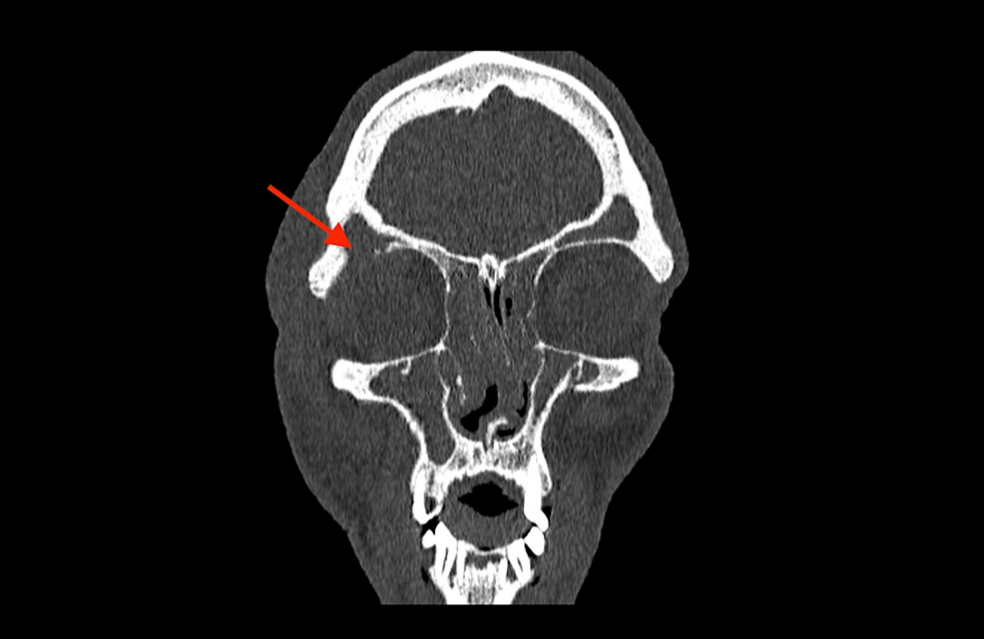
Computed tomography (CT): a coronal reconstruction with bone windows showing complete opacification of bilateral maxillary, ethmoid and frontal sinuses with a defect in the inferolateral aspect of the right frontal sinus

**Figure 2:**
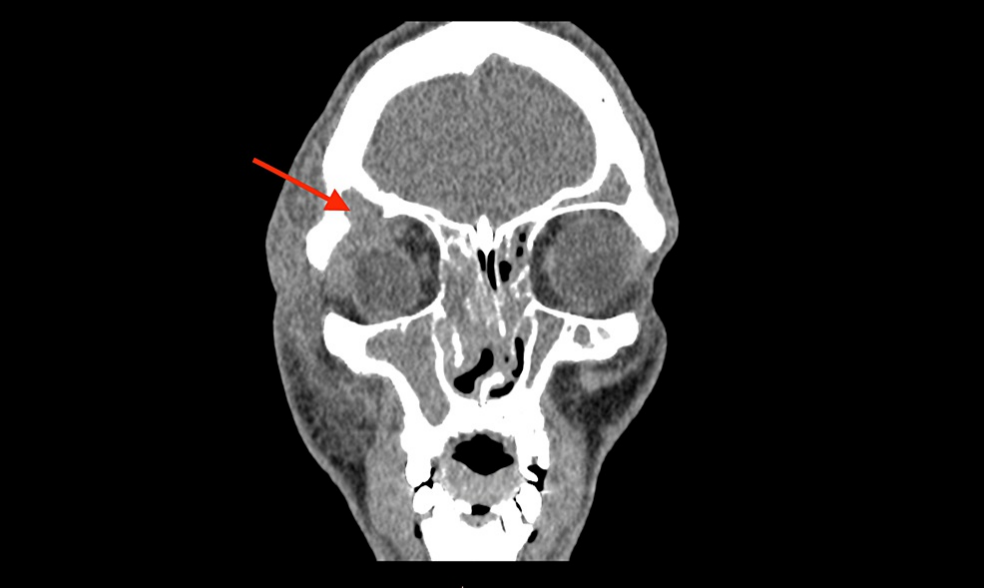
The same computed tomography (CT) slice with soft tissue windowing demonstrating soft tissue signal (the mucocoele) extending from the right frontal sinus to the superolateral aspect of the right orbit with consequent displacement of the orbit and associated more diffuse soft tissue swelling

A computed tomography (CT) scan of the head was performed due to the patient’s recurrent symptoms, which showed complete bilateral opacification of the frontal, anterior ethmoid and maxillary sinuses with osteomeatal complex obstruction. There was an 11 mm bony defect in the superolateral aspect of the right orbit (Figure 1) with herniation of soft tissue density from the right frontal sinus to the superior aspect of the right orbit associated with periorbital soft tissue swelling (Figure 2).

The patient was referred to otolaryngology for review. On further questioning, he reported a three-year history of nasal obstruction and rhinorrhoea. He was admitted and commenced on intravenous ceftriaxone and metronidazole as per microbiology advice alongside intravenous dexamethasone and topical nasal decongestants. He was scheduled for emergent surgical intervention to drain the mucopyocoele and restore the patency of the frontal drainage pathway; however, he responded well to medical management, and surgery was deferred.

Functional endoscopic sinus surgery (a frontal sinusotomy: Draf IIa) was performed semi-electively a week later. The presence of the likely longstanding mucopyocoele was evident intraoperatively, and after endoscopic drainage, it was possible to wash out the sinus with a large-calibre needle passed through the bony dehiscence beneath the lateral orbital rim. The patient was discharged the same day and has remained well postoperatively with no recurrence of sinonasal or periorbital symptoms.

## Discussion

It is important in patients presenting with signs suggestive of periorbital cellulitis to identify the primary cause of the pathology and not just manage the cellulitis itself. 

Periorbital cellulitis mostly arises following the spread from an acute infective source, which may resolve spontaneously or with the cellulitis treatment. However, in this case, a chronic process (frontal mucocoele) was present, most likely with an initial infection of the mucocoele (becoming a mucopyocoele) and then spreading very easily to a periorbital cellulitis. A chronic persistent infection (chronic mucopyocoele) that episodically progressed to periorbital cellulitis is a possible explanation also.

His frontal mucocoele itself had likely developed over a prolonged period as a result of untreated sinus pathology. Chronic inflammation, whether secondary to infection, allergy or any other causes of poor drainage/mucociliary clearance, can lead to both nasal polyp formation and mucocoeles [5]. Mucocoeles in the paranasal sinuses can expand both intra-orbitally and intracranially if left untreated [6].

Odontogenic sinus mucocoeles are also well described, so poor dentition may have played a part. A ruptured periapical cyst within the floor of the maxillary sinus can lead to a chronic mucopyocoele, with infection spreading from the tooth to the maxillary sinus. Over many months, the sinus expands (medially) to obstruct the frontal drainage pathway and create a combined maxillary and frontal mucopyocoele.

The early management of this patient’s sinonasal symptoms (e.g. intranasal corticosteroids) may have led to improvement in sinus drainage pathways reducing the chance of mucocoele formation and subsequent bony erosion, which ultimately led to the described symptoms. Increased awareness of the symptoms and the effective management of sinus pathology are needed both amongst the public and in primary care.

Furthermore, in patients presenting with periorbital cellulitis, a multidisciplinary approach is important to ensure the optimal treatment of the patient. In established mucocoeles and mucopyocoeles, functional endoscopic sinus surgery is the definitive contemporary treatment, establishing drainage and facilitating wash-out and medication delivery without the need for external scars [6]. In less experienced hands and centres with less modern facilities, external approaches to achieve the same drainage remain valid.

## Conclusions

A frontal mucopyocoele is an uncommon but important differential diagnosis when a patient presents with symptoms of periorbital cellulitis. A full clinical assessment should incorporate a sinonasal history and examination with imaging, where indicated. We suggest that patients presenting with recurrent periorbital cellulitis with no other clear cause should undergo imaging (a CT scan of the paranasal sinuses).

Mucocoeles can lead to the expansion and erosion of bony structures leading to complications within neighbouring structures such as the orbit or cranial vault, especially in the context of infection (mucopyocoeles), either chronic or recurrent. The identification of such underlying triggers in periorbital cellulitis can expedite medical and surgical management, preventing complications and morbidity.
